# Investigating the Use of Circulating Tumor DNA for Sarcoma Management

**DOI:** 10.3390/jcm13216539

**Published:** 2024-10-31

**Authors:** Paige Darville-O’Quinn, Nalan Gokgoz, Kim M. Tsoi, Irene L. Andrulis, Jay S. Wunder

**Affiliations:** 1 Lunenfeld-Tanenbaum Research Institute, Sinai Health System, Toronto, ON M5G 1X5, Canada; paige.darvilleoquinn@gmail.com (P.D.-O.); nalan@lunenfeld.ca (N.G.); kim.tsoi@sinaihealth.ca (K.M.T.); 2 Musculoskeletal Oncology Unit, Sinai Health System, University of Toronto, Toronto, ON M5S 1A1, Canada; 3 Department of Surgery, University of Toronto, Toronto, ON M5S 1A1, Canada; 4 Department of Molecular Genetics, University of Toronto, Toronto, ON M5S 1A8, Canada; 5 Department of Laboratory Medicine and Pathobiology, University of Toronto, Toronto, ON M5S 1A8, Canada

**Keywords:** cell-free DNA, circulating tumor DNA, ctDNA, sarcoma, soft tissue sarcoma, biomarkers, ddPCR, multiplex PCR, liquid biopsy

## Abstract

**Background/Objectives**: Sarcomas are a heterogeneous group of cancers, many with high rates of recurrence and metastasis, leading to significant morbidity and mortality. Due to a lack of early diagnostic biomarkers, by the time recurrent disease can be clinically detected, it is often extensive and difficult to treat. Here, we sought to investigate methods of detecting ctDNA in sarcoma patient plasma to potentially monitor disease recurrence, progression, and response to treatment. **Methods**: Whole-exome sequencing of matched tumor and blood samples revealed patient-specific mutations, which were used to develop personalized assays to detect ctDNA in patient plasma. Since ctDNA is present in extremely low quantities, detection requires highly sensitive methodologies. Droplet digital PCR is highly sensitive; however, it is limited in that it can only be used to target one tumor variant at a time. Therefore, a protocol combining multiplex PCR and targeted amplicon sequencing was developed. **Results**: ddPCR was successfully able to detect tumor-specific mutations in plasma, confirming the presence of ctDNA in sarcoma patients. Multiplex PCR followed by amplicon sequencing was able to detect multiple tumor variants simultaneously, although it was not as sensitive as ddPCR. Additionally, ctDNA was detected in patient plasma collected at two different time points. **Conclusions**: This work demonstrates that although there is a lack of recurrent biomarkers, personalized assays detecting ctDNA have the potential to be used to monitor disease progression in sarcoma.

## 1. Introduction

Sarcomas are a rare and diverse group of cancers arising from mesenchymal tissues. They account for approximately 1% of adult and 20% of pediatric malignancies and 2% of cancer-related deaths [1]. As cancers of mesenchymal tissues, sarcomas are categorized broadly as originating from either bone or soft tissue, and there are more than 80 distinct types [2]. According to the World Health Organization’s 2020 Classification of Soft Tissue and Bone Tumours, soft tissue sarcomas (STS) can be further subdivided based on the site of origin, for example extremity vs. retroperitoneal, and also by the tissue of origin [3]. Examples include adipocytic tumors, such as liposarcoma, fibroblastic tumors, such as myxofibrosarcoma, vascular tumors, such as angiosarcoma, smooth muscle tumors, such as leiomyosarcoma, tumors of uncertain origin, such as undifferentiated pleomorphic sarcoma, and more. Osteosarcoma is the most common histological type of bone sarcoma [4]. Osteosarcomas can develop in any bone; however, the majority arise in the long bones of the extremities [3]. Bone and soft tissue sarcomas are highly heterogeneous, making the field of sarcoma research challenging since an understanding of one subtype may not translate to another [2].

The main treatment for patients with sarcoma is wide surgical resection, with the goal of complete tumor removal [1]. Many patients with soft tissue sarcoma (STS) receive radiation therapy either pre-operatively or post-operatively to eliminate microscopic disease surrounding the primary tumor. In comparison, chemotherapy is used most commonly in patients with bone sarcomas to target microscopic metastatic disease. Complete surgical resection can be curative; however, within 3 years of the initial surgery 40–60% of sarcoma patients will develop either local recurrence or distant metastases [5]. Once metastases occur, the survival rate drops to less than 15% [1]. Currently, there are no molecular biomarkers for the detection of locally recurrent or metastatic sarcoma, and identification is left to identifying a palpable mass clinically and radiographic imaging, including MRI, CT, and PET scans [6]. By the time recurrent disease can be visualized through diagnostic imaging, it is often extensive and difficult to treat [7]. In many cases, if surgery, radiation, and chemotherapy have failed there are few effective treatment protocols remaining [8].

In recent years, there has been growing interest in the use of circulating tumor DNA (ctDNA) as a biomarker for monitoring disease progression in patients with various cancers [7]. DNA that is released into the bloodstream through necrosis and apoptosis is referred to as cell-free DNA (cfDNA) [9]. This DNA is highly degraded and is present in short fragments, approximately 170 base pairs in length [9]. In healthy individuals, cfDNA is derived predominantly from hematopoietic cells. Circulating tumor DNA, however, is a type of cell-free DNA that is released specifically by tumor cells [9]. While mean cfDNA levels in healthy individuals range from 3 to 7 ng per mL of plasma, they are often elevated in cancer patients [10]. The amount of ctDNA in the blood corresponds with the tumor burden; however, cfDNA levels are also affected by factors, such as exercise and infection [11]. Mutations in ctDNA correspond to mutations from the primary tumor; thus, a “liquid biopsy” has the potential to capture the genetic landscape of highly heterogeneous tumors without requiring a surgical procedure to obtain actual tumor tissue [1,11,12]. Because it is easier to obtain, blood can be collected at multiple time points, thus providing important information, such as disease progression and response to treatment over time.

One of the main challenges to using ctDNA as a biomarker is that it is present in extremely low quantities, in some cases making up less than 0.1% of the overall cfDNA [13]. Thus, extremely sensitive methods of detection are needed, which require prior knowledge of alterations in the tumor genome. This creates an additional challenge for studying sarcomas as there is a lack of hallmark mutations common across all tumor cases [14]. Some sarcoma subtypes, such as chondrosarcoma and Ewing sarcoma, have recurrent genomic alterations, which can be used for ctDNA detection [15,16]. However most adult soft tissue sarcoma subtypes lack any such recurrent mutations [17].

Due to its high sensitivity, droplet digital PCR (ddPCR) can be used to detect ctDNA. Our group has successfully used ddPCR to detect patient-specific tumor mutations in cfDNA samples, thus confirming the presence of ctDNA in sarcoma patient plasma [18]. However, the main disadvantage of ddPCR is its inability to test for multiple mutations at once. The genetic profiles of most sarcomas are highly complex, with multiple different genomic mutations and alterations. Therefore, each patient’s tumor will harbor its own specific mutations in ctDNA. Because the relative abundance of ctDNA is so low, the ability to test for multiple mutations simultaneously would increase the chances for detection in the blood. Unfortunately, this is not feasible using ddPCR as it cannot be multiplexed. As an alternative, multiplex PCR (mPCR) can simultaneously amplify multiple target regions of DNA, thereby allowing multiple tumor-specific mutations to be detected simultaneously using targeted Next Generation Sequencing (NGS).

We previously showed that personalized assays can be used to detect ctDNA in sarcoma patient plasma based on individual, tumor-specific variants using ddPCR [18]. The present study sought to determine whether a combined mPCR-targeted amplicon sequencing methodology would improve the ability to detect ctDNA by seeking out multiple genetic variants at once. Furthermore, the ability to use these assays for disease surveillance was investigated by testing cfDNA samples obtained from the same patients at different time points.

## 2. Materials and Methods

### 2.1. Sample Collection and Processing

Peripheral blood samples were obtained pre-operatively from sarcoma patients who had provided written informed consent under approved institutional ethics board protocols (REB# 01-0138-U). Samples were collected in two 10 mL EDTA tubes and then processed on ice within one hour of collection to prevent hemolysis. Plasma was separated via centrifugation at 2500× *g* for 10 min at 4 °C, transferred to microfuge tubes, and centrifuged again at 16,000× *g* for 10 min at 4 °C to remove cellular debris. The resulting plasma samples were then stored in 1 mL aliquots at −80 °C. Ideally, we attempted to obtain approximately 10 mL of plasma from each patient. However, the quantity and quality of plasma isolated varies on an individual basis. Matched patient tumor samples from open biopsies and/or subsequent surgical resection specimens were also collected and stored.

### 2.2. cfDNA Extraction and Assessment

Cases chosen for cfDNA extraction were selected based on having a high starting volume of plasma with no signs of hemolysis. cfDNA was isolated from 2 mL of plasma using the QIAamp circulating nucleic acid extraction kit (Qiagen, Germantown, MD, USA) with the QIAvac 24 Plus vacuum manifold (Qiagen, Germantown, MD, USA). Extractions were performed according to the manufacturer’s instructions with the following modifications: no carrier RNA was added to the buffer ACL; the sample-buffer ACL mixture was incubated at 60 °C for 60 min; DNA columns were eluted with 60 μL of buffer AVE; samples were incubated for 6 min after the buffer AVE was applied to the membrane; the eluate was reapplied to the column following the first elution, incubated, and eluted a second time via centrifugation. Extracts were then stored at −80 °C.

The concentration of extracted cfDNA was determined via RT-qPCR using an 81 bp amplicon of the *EIF2C1* gene on chromosome 1 and a dilution series of placenta DNA. RT-qPCR assays were performed using the Power SYBR Green PCR Master Mix (Applied Biosystems, Waltham, MA, USA) on the CFX96 Real Time PCR Detection System (Bio-Rad, Hercules, CA, USA). To assess cfDNA quality, samples were speed vacuumed and diluted with distilled water to achieve a concentration of 1 ng/μL. These samples were then submitted for capillary electrophoresis (CE) analysis using the 2100 Bioanalyzer (Agilent Technologies, Santa Clara, CA, USA). In total, cfDNA was extracted and quantified from more than 60 patients at various time points, 50 of which have also been assessed via capillary electrophoresis.

### 2.3. Identification of Tumor-Specific DNA Variants

DNA was extracted from freshly frozen tumor samples and matched blood using the DNeasy Blood and Tissue Kit (Qiagen, Germantown, MD, USA), according to the manufacturer’s instructions. Whole-exome sequencing (WES) was conducted on twelve matched tumor-whole blood pairs. Cases were selected for sequencing based on tumor viability, as well as the quantity and quality of corresponding cfDNA, as determined based on the results of qPCR, and CE WES was performed using the Illumina HiSeq 2500 (2 × 125 bp) (Illumina, San Diego, CA, USA) platform with 70–100× average coverage. Sequencing was conducted by The Centre for Applied Genomics, The Hospital for Sick Children, Toronto, Canada. Mutect2 analysis was then conducted for somatic variant calling. Mutect2 uses local assembly and realignment to detect SNVs and indels that are present in the tumor DNA and not the matched normal blood DNA [19].

### 2.4. Droplet Digital PCR

#### 2.4.1. Target Selection and Assay Design

Primers and allele-specific probe sets were designed based on the WES results of five cases (Table 1 and Table 2). For each case, the tumor-specific SNV with the highest allele frequency, as identified via sequencing, was chosen to be used as a target for detection via ddPCR. ddPCR primers and probes were designed and then synthesized by Integrated DNA Technologies (IDT) Canada Inc. (IDT Canada, Ottawa, ON, Canada) The ddPCR assays were performed using the QX200™ AutoDG™ Droplet Digital™ PCR System (Bio-Rad, Hercules, CA, USA). Assays were first tested using a temperature gradient between 50 °C and 60 °C, using Hexachloro-fluorescein (HEX) and 6-carboxyfluorescein (FAM) intercalating fluorophores for wild-type and mutant alleles, respectively.

#### 2.4.2. ddPCR Sensitivity Assays

Prior to testing the cfDNA samples, sensitivity assays were conducted using tumor and blood DNA to determine the limits of detection of mutant DNA for each case. These assays have previously been described by our group, as well as others [18,20]. The first sensitivity assay was performed by serially diluting the tumor DNA in water such that the concentrations of DNA analyzed via ddPCR were between 20 ng and 0.25 ng. The goal of this assay was to determine the minimum amount of input DNA needed to detect a mutation.

The second sensitivity assay was performed by serially diluting the tumor DNA in normal blood DNA to investigate the limits of detecting a low mutant allele fraction amongst a wild-type background. This imitates the conditions when trying to detect a small amount of ctDNA amongst wild type cfDNA. Here, the samples contained between 100% and 0.5% tumor DNA.

#### 2.4.3. cfDNA Assays

cfDNA assays were conducted according to previously optimized ddPCR conditions. The detection of tumor-specific SNVs in cfDNA samples indicated the presence of ctDNA in patient plasma. For each case, between 5 ng and 15 ng of cfDNA was utilized for ddPCR.

### 2.5. Multiplex PCR and Targeted Amplicon Sequencing

#### 2.5.1. Multiplex PCR Primer Design

WES analysis identified patient-specific genetic alterations in tumors. For each case, the list of variants was narrowed to the top 15 most promising candidates for mPCR, with priority given to variants with a high allele frequency, variants present in cancer related-genes according to the COSMIC database, and variants known to alter the protein coding sequence.

Primers were designed using PrimerBlast (NCBI, Bethesda, MD, USA) according to the following parameters: amplicons generated must be between 60 and 150 bp, primer length between 18 and 25 nt, primer melting point (Tm) approximately 60 °C. NetPrimer software version 1.10 (Premier Biosoft, San Francisco, CA, USA) was used to ensure that primer pairs would allow for secondary structure or dimer formation, and additionally ThermoFisher’s (Waltham, MA, USA) Multiple Primer Analyzer was used to check for any potential cross-reactivity between primer sets in the multiplex. Any primer pairs with the potential for off-target amplification or dimer formation were removed from consideration. As a result, for each case, between 6 and 8 primers were ordered from Eurofins Genomics (Luxembourg City, Luxembourg) (Table 3).

#### 2.5.2. Multiplex PCR Optimization

Primers were first tested under singleplex conditions to ensure that they worked appropriately. A temperature gradient was conducted to select a universal annealing temperature at which the primers sets were combined in multiplex.

In the initial testing of primers pairs in multiplex, each primer was added to the reaction in an equal concentration. However, the optimization of primer amounts was required so that each target region was amplified with the same efficiency. The final concentration of each primer in the 50 μL mPCR reaction ranged from 0.1 μM to 2 μM. All PCR assays were conducted using Phusion Hot Start II High-Fidelity PCR Master Mix (Thermo Scientific, Waltham, MA, USA).

#### 2.5.3. Multiplex PCR Amplicon Sequencing

Primers and reaction conditions were optimized for four cases, and amplicons were generated via mPCR using patient tumor, blood, and cell-free DNA. Resulting amplicons were sequenced using the Illumina Nextseq (2 × 150 bp) platform by the Sequencing Facility at Mount Sinai Hospital’s Lunenfeld–Tanenbaum Research Institute.

Linearity and sensitivity were assessed using serially diluted tumor DNA mixed with the corresponding blood DNA. The observed variant frequency was then compared to the expected frequency calculated based on the variant allele frequency observed in WES.

A second round of sequencing was performed using the Illumina NovaSeq (2 × 150 bp) platform by The Centre for Applied Genomics, The Hospital for Sick Children, Toronto, Canada.

## 3. Results

### 3.1. Analysis of cfDNA Extracted from Plasma

**Figure 1:**
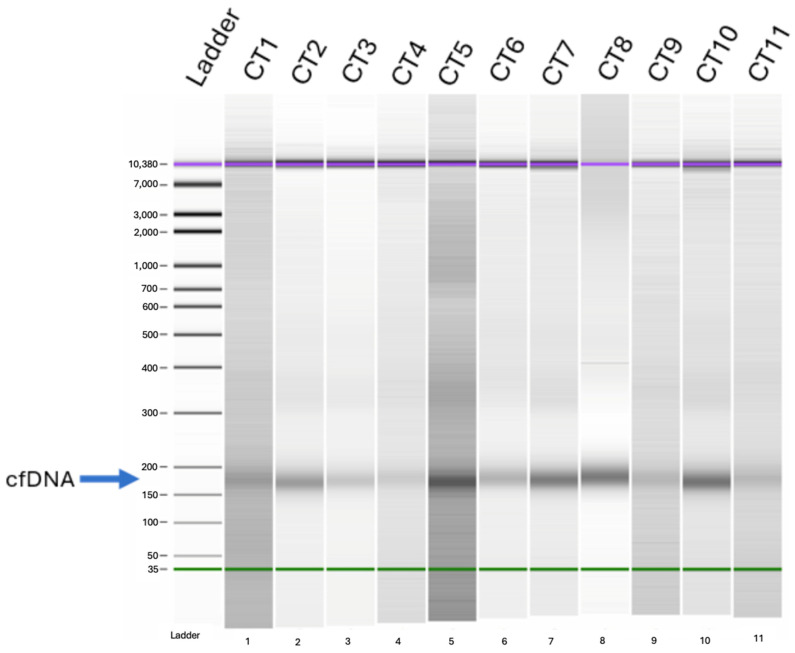
cfDNA quality assessment via capillary electrophoresis. DNA isolated from plasma samples was separated based on the fragment size. Lane L contains a DNA ladder, and lanes 1–11 contain extracted DNA. Samples have signal peaks corresponding to DNA fragments of 150–200 bp, which is the range consistent with cfDNA. Purple line corresponds to the highest size on the DNA ladder, green line corresponds to the lowest size on the DNA ladder.

Over 60 patients were selected for cfDNA extraction based on high starting volumes of plasma with no signs of hemolysis. qPCR and capillary electrophoresis (CE) were conducted on cfDNA extracts to determine the concentration of DNA in each sample, as well as the quality of DNA extracted. CE separates DNA fragments based on size with a high degree of sensitivity, thus revealing any contamination. cfDNA, due to its fragmented nature, will be present at approximately 170 bp, and any other bands are indicative of genomic DNA (Figure 1). All tested samples were positive for signal peaks representing cfDNA.

### 3.2. Target Selection for Patient-Specific cfDNA Detection Assays

Whole-exome sequencing of tumor and blood pairs for twelve sarcoma patient cases revealed a wide variety of somatic alterations. Of these, four cases were selected for the development and optimization of mPCR-targeted amplicon sequencing assays. These cases were chosen because the tumor variants identified through WES had a relatively high allele frequency and read depth, the majority of alterations were point mutations that impacted the protein-coding sequence (e.g., non-synonymous SNVs, stop-gain mutations), and having a high concentration of corresponding cfDNA. For each case, mPCR primer pairs were designed for 6–8 variants (Table 4) In general, the variants with the highest allele frequency were selected as mPCR targets, as well as those that altered the protein coding sequence whenever possible. Cases were concurrently analyzed via ddPCR to confirm mPCR amplicon sequencing results, as we previously established that this technique can detect ctDNA [18]. For each case, one of the non-synonymous single nucleotide variants (SNVs) identified via sequencing was selected as a target (Table 4).

### 3.3. ddPCR Is Highly Sensitive

**Figure 2:**
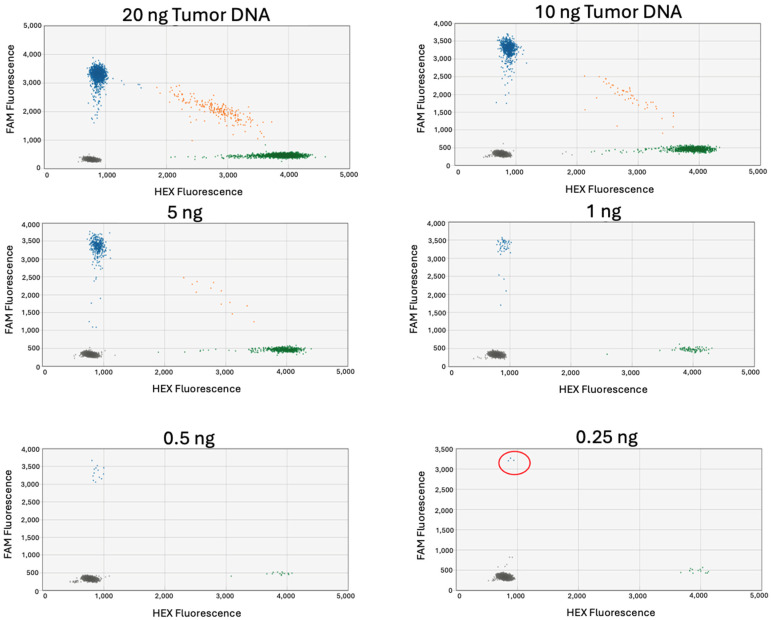
Tumor DNA diluted in water. ddPCR conducted on serial diluted samples of tumor DNA in water from 20 ng down to 0.25 ng. In these figures, the *x*-axis represents the intensity of the HEX fluorescence (corresponding to the wild-type probe), and the *y*-axis represents the intensity of the FAM fluorescence corresponding to the mutant probe). Thus, the green cluster represents the population of droplets containing the wild-type allele, and the blue cluster represents the population of droplets containing the mutant variant allele. The orange cluster represents the double positive population meaning that the droplets contained both the mutant and wild-type allele. Variant allele can be detected with as little as 0.25 ng tumor DNA as indicated in the red circle. The case used in this example is RT-7418; primers and probes targeted an SNV in the gene SEMA5A.

The goal of ddPCR was to detect tumor-specific mutations amongst the higher proportion of the wild-type versions of the genes. Before testing the cfDNA extracts for the presence of ctDNA, the sensitivity of each probe was analyzed. Two types of sensitivity assays were conducted, each involving serial dilutions of tumor DNA. First, tumor DNA was diluted in water to determine the minimum amount of input DNA required to detect a mutation. Here, 20 ng of tumor DNA was diluted in water so that the input DNA used in ddPCR was between 20 ng and 0.25 ng (Figure 2). ddPCR is highly sensitive and in all cases tested, the variant allele was detected with as little as 0.25 ng of input tumor DNA.

**Figure 3:**
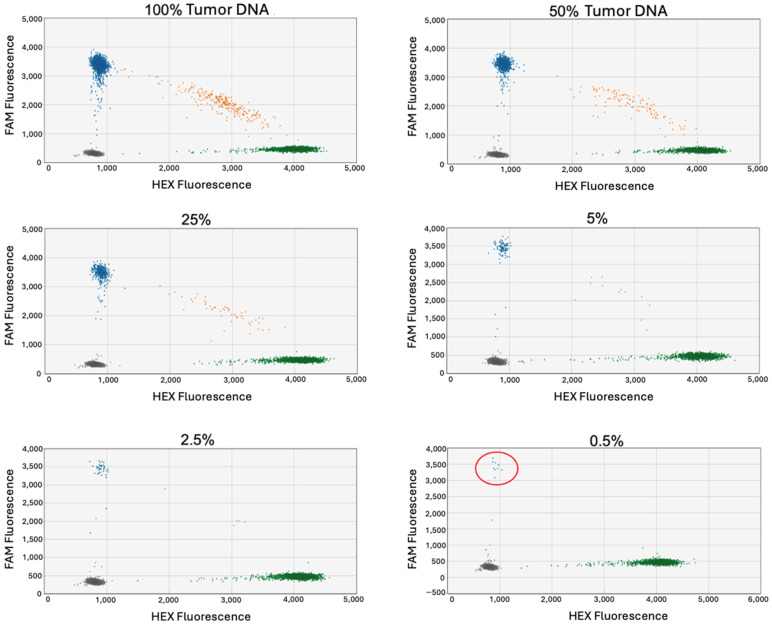
Tumor DNA diluted in blood DNA. ddPCR conducted on serial diluted samples of tumor DNA in blood DNA from 100% tumor DNA down to 0.5% tumor DNA. Case RT-7418; primers and probes targeted an SNV in the gene SEMA5A. Variant allele can be detected with as little as 0.5% tumor DNA as indicated in the red circle.

Next, tumor DNA was diluted in blood DNA to determine whether the mutant allele could be detected when only present as a very small percentage compared to the wild-type allele. Samples tested ranged from 100% tumor DNA to 0.5% (Figure 3). In these tests, while the mutant concentration progressively decreases, the wild-type concentration increases. Thus, this assay more closely represents the conditions of testing cfDNA samples in which ctDNA harboring the tumor variant will be present in very small proportions compared to a larger wild-type background.

In all cases, the variant allele could be detected when the tumor DNA comprised as little as 0.5% of the input DNA sample. The results of this assay across all cases tested demonstrated ddPCR to be highly sensitive. Because the conditions were designed to approximate ctDNA, this assay showed that ddPCR has the capacity to detect ctDNA.

### 3.4. mPCR Optimization

**Figure 4:**
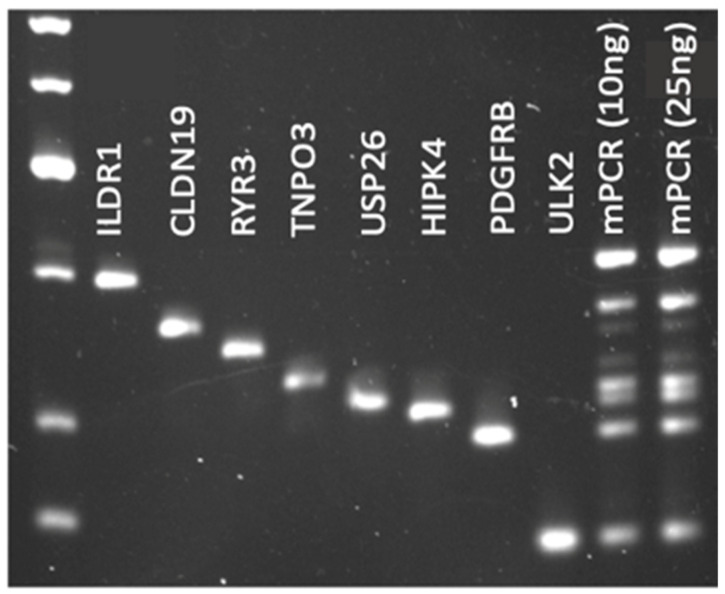
Unoptimized mPCR conditions. Agarose gel depicting mPCR for case RT-7860. Lane 1 contains the DNA Ladder. Lanes 2–9 contain the individual amplicons from singleplex PCR. Lanes 10 and 11 contain the mPCR product using either 10 ng or 25 ng of input DNA, where primers were added to the reaction mix in an equal concentration.

**Figure 5:**
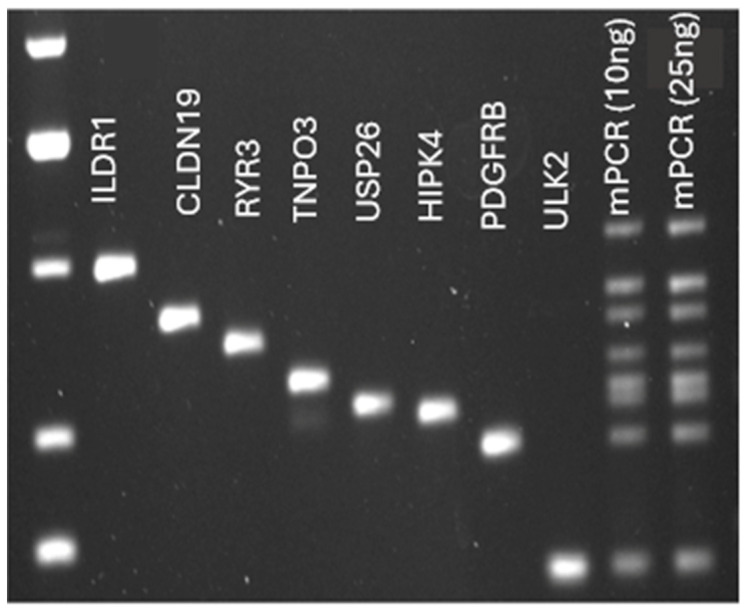
Optimized mPCR conditions. Agarose gel depicting mPCR for case RT-7860. Lane 1 contains the DNA ladder. Lanes 2–9 contain the individual amplicons from singleplex PCR. Lanes 10 and 11 contain the mPCR product using either 10 ng or 25 ng of input DNA, after optimizing the primer amount and reaction conditions for this case. Here, each sequence was amplified with equal intensity.

To begin, primers were designed to amplify eight tumor variants identified via WES per case. Primers were first tested individually across a temperature gradient, and at this point, any primers that resulted in dimer formation or off-target amplification were excluded. The temperature gradient was run to test a variety of annealing temperatures to ensure that the mPCR conditions would work for all primer sets simultaneously. Once the primer sets were verified, mPCR conditions were optimized. Due to differing primer efficiencies, some sequences were potentially amplified with a differing intensity (Figure 4). To rectify this, the ratio of different primers was adjusted until each amplicon appeared in an agarose gel with a similar intensity (Figure 5). Again, if any primer dimers appeared at this stage of optimization, they were excluded.

### 3.5. Targeted Amplicon Sequencing

**Figure 6:**
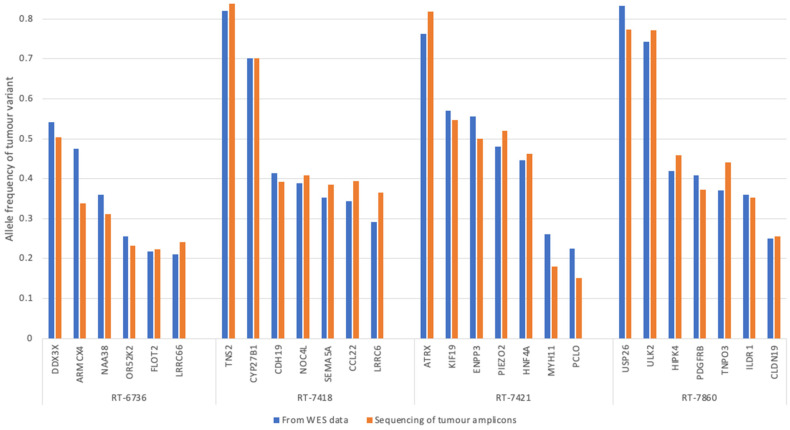
Tumor variant sequences detected via mPCR-amplicon sequencing. Results of sequencing the amplicons generated from tumor DNA for all cases show that the variant allele frequencies from WES and the targeted amplicon sequencing were similar. The results are based on the detection of variant alleles that were identified via WES of the tumor DNA. The *x*-axis shows each amplicon sequence, and the *y*-axis is the allele frequency of the tumor variant. The blue bars represent the variant allele frequency as determined via the whole-exome sequencing of tumor and blood DNA; the orange bars represent the frequency of the tumor variant in the mPCR products generated from tumor DNA.

Multiplex PCR amplicons were generated using tumor DNA and sequenced with the goal of detecting the tumor variants previously identified via WES of matched tumor-whole blood DNA pairs. First, to determine whether the amplicon sequencing was accurate, the variant allele frequencies obtained through sequencing the tumor amplicons were compared to those observed in the previous WES data (Figure 6).

Each of the amplicons generated from tumor DNA was able to detect tumor variant sequences and at a frequency similar to that observed in WES. This indicates that mPCR successfully amplified the sequences of interest in the tumor DNA and that sequencing results were accurate. Next, mPCR was also conducted on DNA samples generated by serially diluting tumor DNA in blood DNA to assess assay sensitivity. The mPCR products from this dilution series contained 100%, 50%, 25%, 5%, 2.5%, or 0.5% tumor DNA. Across all cases, the majority of variants could be detected when the tumor DNA made up 5–2.5% of the sample. Only one amplicon was detected at the lower tumor DNA percentage of 0.5%. In general, the variants that could not be detected in dilutions below 25% were those with lower variant allele frequencies within the bulk tumor DNA.

### 3.6. ctDNA Can Be Detected Through Both ddPCR and mPCR-Amplicon Sequencing

The variant alleles were detected by sequencing cfDNA amplicons in two out of four cases (Table 5). The wild-type alleles only were detected in the cfDNA of the remaining two cases.

**Figure 7:**
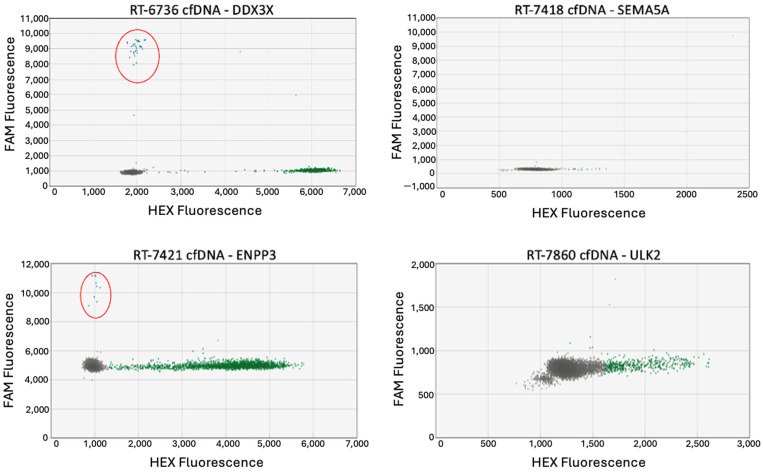
ctDNA detection via ddPCR. ddPCR was conducted on cfDNA samples for each case. The tumor variant was detected in two of the four cases. The red circled clusters contain the variant allele.

To confirm whether the results of cases RT-7418 and RT-7860 were due to a lack of ctDNA in those two cases or whether the mPCR-amplicon sequencing protocol was not sufficiently sensitive to detect the tumor-specific variants, one of the nonsynonymous SNVs was selected as a target for ddPCR assay design (Figure 7). Because the chosen variants, *SEMA5A* for RT-7418 and *ULK2* for RT-7860, could not be detected in cfDNA via ddPCR, it is likely these cases were indeed negative for ctDNA.

### 3.7. ddPCR Can Detect ctDNA in Plasma Obtained at Multiple Time Points

For some patients, blood was collected at various time points and plasma was isolated from each specimen for cfDNA detection. As previously described, DNA from the primary tumor tissue and whole blood were sequenced and compared to reveal tumor-specific variants. For one case in particular, RT-7707, plasma was collected during an initial biopsy, as well as one month later at the time of definitive surgical resection. cfDNA was extracted from plasma obtained at both time points. ddPCR primers and probes were designed to detect one of the nonsynonymous SNVs previously identified via the WES of primary tumor DNA—in this case *TTLL5*. Primers and probes were tested across a temperature gradient to optimize ddPCR conditions, followed by sensitivity assays as previously detailed.

**Figure 8:**
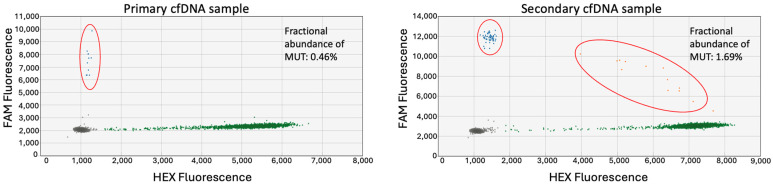
Detection of ctDNA in paired cfDNA samples. RT-7707 cfDNA collected at two separate time points, evaluated via a ddPCR assay targeting a primary-tumor-specific variant in the gene *TTLL5*. The red circled clusters contain the variant allele.

Once confident in the RT-7707 ddPCR assay, the plasma cfDNA samples were tested (Figure 8). A total of 5 ng of DNA was used for each assay, and the tumor variant was detected in the cfDNA in both samples indicating that ctDNA was present in the plasma collected at both time points. Moreover, the fractional abundance of the mutant allele was increased in the secondary cfDNA sample, indicating that there is more tumor DNA present in the later sample.

### 3.8. Sarcoma Type, Disease Stage, Tumour Burden, and Metastatic Status Influence ctDNA Detection

In total five cases were analyzed via ddPCR, of which three were found to be positive for ctDNA. To determine what factors may influence the ability to detect ctDNA in patient plasma, the clinical features of each tumor were compared. The goal was to determine how tumor size and grade, metastatic status, and sarcoma subtype impacted the detection of ctDNA (Table 6). In general, the higher the disease burden, the more likely it was for ctDNA to be identified in the bloodstream. All three positive cases had high-grade sarcomas associated with metastatic disease.

## 4. Discussion

Quantitation via qPCR often found the concentration of cfDNA extracted from sarcoma patients to be low. Since circulating DNA is not limited to tumor DNA alone and also contains DNA released by normal cells, extremely sensitive methods are necessary to detect ctDNA.

Assays conducted using tumor DNA confirmed ddPCR’s ability to detect variants present in extremely low quantities. In our pilot study and in additional cases described here in this report, the variant allele was detected via ddPCR with as little as 0.25ng of input tumor DNA [18]. When diluted with blood DNA to better approximate the conditions of detecting ctDNA within a cfDNA sample, the variant allele could be detected when the tumor DNA comprised as little as 0.5% of the input DNA sample across all cases. Together, these results demonstrate that ddPCR is highly sensitive in accurately detecting tumor DNA in extremely low quantities. This sensitivity is important when considering using ctDNA assays for follow-up surveillance post-operatively. At diagnosis, depending on factors, such as the disease stage, tumor size, and type of sarcoma, up to 90% of DNA in the circulation may be tumor-derived [10,21]. However, following surgery, ctDNA levels drop significantly [10]. ctDNA assays must therefore be able to detect tumor variants even when they are present in extremely low quantities [22].

Although ddPCR is sensitive and capable of detecting ctDNA in the plasma of sarcoma patients, a potential limitation is its inability to be multiplexed. mPCR followed by amplicon sequencing circumvents this issue and provides an alternative method for detecting ctDNA based on the simultaneous analysis of multiple tumor variant sequences. To assess the accuracy of the mPCR-amplicon sequencing protocol, the allele frequencies of the tumor variants of interest in the DNA amplicons were compared to the previous whole-exome sequencing results. The detection of variant sequences in the tumor amplicons at a frequency comparable to that of WES indicates that when sequences are amplified via mPCR, the proportions of the mutant and wild-type alleles are preserved, and the sequencing results remain accurate.

ddPCR and mPCR-amplicon sequencing were both used to develop personalized detection assays for four sarcoma patients, and ctDNA was detected using both methods in two of four cases. While this demonstrates that they each have the ability to detect ctDNA, there are also limitations to each method. As previously discussed, the finite amount of cfDNA available for each case restricts the number of tumor variants that can be targeted via ddPCR. For case RT-6736, only one variant was analyzed via ddPCR, while six were assayed through mPCR-amplicon sequencing. Of the six sequences amplified via mPCR, the tumor variant was detected in only three. The remaining three only identified wild-type sequences; consequently, if one of those variants had been selected for a ddPCR assay, it would have yielded a false negative result. Thus, by broadening the number of gene sequences analyzed at the same time, mPCR-amplicon sequencing improves the accuracy of detecting ctDNA. However, mPCR-amplicon sequencing is not as sensitive as ddPCR. To test the lower limits of detection for tumor variants via mPCR-amplicon sequencing, mPCR was conducted on DNA samples generated by serially diluting tumor DNA in blood DNA—similar to previously described ddPCR sensitivity assays. Most variants were detected when the tumor DNA made up between 5% to 2.5% of the sample, but no lower. In contrast, ddPCR detected variants even when the tumor DNA comprised as little as 0.5% of the sample.

Due to the high rates of tumor recurrence and particularly metastasis in sarcoma patients, an important application of ctDNA detection assays would be for disease surveillance. In the case of one patient, ctDNA was detected via ddPCR in plasma samples obtained at two different time points approximately one month apart. The primary cfDNA was collected when the patient had a diagnostic biopsy, and the secondary cfDNA was collected when the patient returned for surgical resection. The concentration of cfDNA extracted from these two sets of plasma samples increased between biopsy and resection. This elevation of cfDNA level could be related to the tumor burden and tumor progression as it was large, high grade, and had evidence of local tissue invasion histologically and metastasis at diagnosis. While cfDNA levels do rise in response to disease progression, it may be unlikely that such an increase in the tumor burden would occur over only one month’s time. However, there are other factors that could have contributed to the increase in the cfDNA concentration. Biopsies are associated with trauma and necrosis of the tumor tissue, which increases the amount of tumor DNA shedding into circulation [23]. In addition, biopsies can result in swelling in the tissues surrounding the tumor, which can also lead to an overall increase in cfDNA [23].

There are many factors that impact the ability to detect ctDNA in patient plasma. In general, concentrations of ctDNA may correspond to the tumor burden [24]. Thus, the bigger the tumor and the more advanced the disease stage, the more ctDNA may be present in the plasma. Other factors that influence ctDNA levels include the extent of tumor infiltration, whether the tumor is necrotic, and the magnitude of tumor vascularity [25]. Because cfDNA is released by apoptotic and necrotic cells, higher-grade tumors with more necrotic tissue may release more tumor DNA into the circulation. Furthermore, deep tumors and tumors that are highly vascularized may result in higher levels of ctDNA as they have easier access to the bloodstream [25]. Metastatic tumors also have a higher likelihood of having detectable levels of ctDNA as they have already extravasated and invaded new areas in the body and, as a result, frequently have a higher tumor burden and are more biologically aggressive [26]. In general, even in patients with high-grade tumors, sarcomas release lower levels of cfDNA compared to other solid cancers [27] Finally, in sarcomas, another factor to consider is the histologic subtype, as some sarcomas are naturally more aggressive than others and have different likelihoods of tissue invasion and metastasis. Both of the cases lacking detectable ctDNA were of a lower grade. RT-7418 was grade 1 and RT-7860 was grade 2, and neither presented with metastasis at diagnosis. RT-7418 was a well-differentiated liposarcoma (WDLPS), a subtype that does not metastasize and has limited potential to invade into surrounding tissues [28]. This subtype also tends to be slow growing, which would reduce the amount of DNA available to be released into the bloodstream during cell proliferation compared to more aggressive and faster growing tumors [28]. It is therefore possible that WDLPS tumors will not have detectable levels of ctDNA in the blood, as is supported by our negative results for case RT-7418. In comparison, RT-7860 was a pleomorphic liposarcoma (PLS), which is known to be more aggressive and have high rates of invasion, recurrence, and metastasis compared to WDLPS [29]. Based on the subtype alone, a patient with a PLS tumor would therefore be more likely to have detectable levels of ctDNA in their plasma. However, in the case of RT-7860, it was intermediate (i.e., grade 2) but not high grade (i.e., grade 3) and was a small tumor, so these characteristics likely contributed to the lack of detectable ctDNA.

In contrast, RT-7707, which was positive for ctDNA at two different timepoints, was a myxofibrosarcoma (MFS). MFS tumors are frequently high-grade with internal necrosis and are biologically aggressive, with high rates of recurrence and metastasis, making it a subtype likely to release ctDNA into the bloodstream [30]. Furthermore, the present MFS case had a high tumor burden in that it was grade 3, very large, with a diameter of approximately 25 cm, exhibited invasion into the adjacent soft tissues radiologically and histologically, and was associated with metastasis at diagnosis. All of these factors likely contributed to the higher levels of ctDNA in the bloodstream at the time of surgical resection compared to the initial biopsy. The remaining cases positive for ctDNA were UPS and osteosarcoma, both of which were also high-grade metastatic tumors. UPS is characterized by an aggressive clinical behavior and has previously been found to have the potential for ctDNA detection [10,18,27,31]. Similarly, osteosarcomas are biologically aggressive tumors that are associated with uncontrolled cellular proliferation and can shed high levels ctDNA into the bloodstream depending on the stage of disease [4,32,33,34]. Our findings support the notion that the presence of ctDNA is dependent on the histological subtype, which also takes into account tumor grade, as well as the overall disease burden based on tumor size and stage.

## 5. Conclusions

Many types of sarcomas have high rates of recurrence and metastasis. Given their aggressive nature, it is conceivable that having a biomarker to survey patients post-operatively would help to detect residual or recurrent disease at an earlier time point than is currently possible based on clinical examination or radiological surveillance and would thereby help guide treatment decisions, including earlier interventions for recurrent or progressive disease. In the present study, we showed that personalized assays are able to detect ctDNA in the plasma of patients with certain types of sarcomas. An ongoing challenge in implementing a liquid biopsy for sarcoma is that recurrent gain-of-function mutations—which are often the basis of ctDNA detection assays in carcinomas—are rare [35]. Tumor-naïve ctDNA panels have been attempted targeting the most commonly mutated genes in soft tissue sarcoma; however, ctDNA detection was limited, and ultimately, this method was abandoned in favor of personalized ddPCR assays, such as that proposed in this research investigation [27]. As a result, tumor-naïve ctDNA assays used for the diagnosis or early detection of recurrent disease are currently not feasible in sarcoma.

This study has demonstrated that personalized assays based on primary tumor-specific mutations can be used to detect ctDNA in the blood of sarcoma patients. Two methods were investigated, ddPCR and mPCR-amplicon sequencing, both of which were able to successfully detect ctDNA. ddPCR was the more sensitive technique, an important consideration for ctDNA detection as it is present in very low quantities; in comparison, mPCR was able to detect multiple tumor variants simultaneously. The personalized assays were also able to detect ctDNA in plasma obtained from a secondary blood draw at a later time point. This shows that ctDNA has the potential to be used for disease monitoring in sarcoma. Specifically, if ctDNA is used to detect molecular residual disease (MRD) in the blood post-operatively, it can influence treatment decisions, including the timing of adjuvant therapy, to potentially improve patient outcomes [24]. A limitation of this study was the finite amount of blood and plasma that could be obtained from each patient. Often there was insufficient cfDNA to conduct requisite replicate experiments. Additionally, not every case investigated had follow-up blood samples. As a result, the majority of cases for which ctDNA was detected were only analyzed at one time point. Despite these inherent limitations in design, these findings are encouraging and warrant future study.

## Figures and Tables

**Table 1:** Forward and reverse primer sequences for all ddPCR targets.

Case	Gene	Forward Primer	Reverse Primer
RT-6736	DDX3X	CCATGTTGATTTCTCCTCAAATTCT	CCCATATCCAACATCCGATCA
RT-7418	SEMA5A	GACACCTGTCAACATCTCTG	TCCCACTTCCAGCAAATTC
RT-7421	ENPP3	ACCTGCCACCAGTTATCT	TGGCATTAAAGTATCCCATGT
RT-7707	TTLL5	AGTTGTGACGATCCAGAAGTG	CGGTTGTATCTCTGCCTTCTT
RT-7860	ULK2	CAGGCTCCTGAGGTTATTATG	TTTTCCAACTAGGCATTGGTA

**Table 2:** Mutant and wild-type probe sequences for all ddPCR targets.

Case	Gene	Forward Primer	Reverse Primer
RT-6736	DDX3X	TTCATCGAACACCA	CTTCATCTAACACC
RT-7418	SEMA5A	TGAGCAATGATTCC	ATGAGCAACGATTC
RT-7421	ENPP3	TTCATCTCTAAATCCATC	TATTCAGCTCTAAATCCA
RT-7707	TTLL5	CATGCTCCATTTTTT	CCATTTGTTTCCAT
RT-7860	ULK2	TCACTGCTCCTATG	TCACTGTTCCTATG

Probe sequences were designed to target the same region with one base pair changed corresponding to the mutant or wild-type sequence.

**Table 3:** All primer sequences used for multiplex PCR assays.

**RT-6736**			
**Amplicon**	**VAF**	**Forward Primer**	**Reverse Primer**
DDX3X	0.542	ACAGCCATACTAAAACCATGTTGAT	AGGCTCAAACCCCATATCCAA
ARMCX4	0.474	TGGCTAGGAATGTGGGAGAG	CTCAGCCCAAAACAAAGACC
NAA38	0.36	AGATGGACGGACACTGGTCG	GCGTAAGGTTCCGCCACA
OR52K2	0.255	TGTTTCCACTACTGCCGAGG	CAAGGAGCAGGTCCAACACC
FLOT2	0.217	TGGTGCAGAGAGATGCTGAC	GCTTCCCATCCTTGAACCCA
LRRC66	0.211	TGGGACTCGCAGATGGAATTT	CACCGTATGGAACCTCGCT
**RT-7418**			
**Amplicon**	**VAF**	**Forward Primer**	**Reverse Primer**
CDH19	0.413	AAGAAAGACCGCTTCACAGAACT	AGAGGAGACGAGCATTATTACCAC
NOC4L	0.389	GACCTTGCTGGCGTATGCTG	GCGGGTGTCGTCGTAGTC
TNS2	0.821	AACCTGGAATCCTAAAGCCGC	TCTCACCAAAGTAGGGCTCAC
SEMA5A	0.353	TCAACATCTCTGACAACGGCG	GGTGCCGTCGCTAGAACAG
CCL22	0.343	TGGTTGTCCTCGTCCTCCTT	GCTCTTGGTCCAGTGCTTGT
LRRC6	0.292	AAAGATAACGAAAAGCAGATCATCC	ACTGAGAATGAAAACAGACAGAAAT
CYP27B1	0.702	GCTCAAGTGCCACCCGAC	GCATCGCCATGGTCAACAGC
**RT-7421**			
**Amplicon**	**VAF**	**Forward Primer**	**Reverse Primer**
ATRX	0.763	TTCTTCTAATCCAAGCAGCCCA	TATCCCCAATTTCCTCTGCCATT
PIEZO2	0.481	CCTGGAGCATCACCTATCACA	TTTCCATAAACCACCATGAAGGGA
ENPP3	0.556	GTTTGACCTGCCACCAGTTATC	GCCACTAGTTACAGAAGACTTACTC
KIF19	0.571	AACCTCCTGAACGTCTCCTAC	CCAGTCTGCTCATCAATCTTGC
MYH11	0.261	CGGGAAAACCGAAAACACCA	CTGCCACTCACCGTGATACT
PCLO	0.224	GATAATTGCCCATGAATCGCTGA	AGAGAAGGTGTGTCAGAGGGT
HNF4A	0.446	ATCCAGGGAAGATCAAGCGG	GTCATACTGGCGGTCGTTGA
**RT-7860**			
**Amplicon**	**VAF**	**Forward Primer**	**Reverse Primer**
ILDR1	0.359	CTCCCATCGCCAATGGTGTC	TTCTGCGTTCCACGACCTCA
CLDN19	0.25	GTGCAGTGCAAGCTCTACGA	CAGCGCAGATAGCAGACTGT
TNPO3	0.37	ATGTGATTGTGTATTTTCTGGCTAT	TAACACTGTAAGGATCTCCAGCAAA
USP26	0.833	CTTGTAAAGCTTGTGGTCAGGTT	TAGACTGAATAGATGAAGGATGTGC
HIPK4	0.419	GGAGTCACACGAACGCATCA	CTGGTAGTAGTGGGTGGTC
PDGFRB	0.409	CTCCGTCCTCTATACTGCCG	CCTCGTCAGCAACCTCGG
ULK2	0.743	ATGCTAAGGCTGACTTGTGG	AAGGTGGTTTTCCAACTAGGCAT

**Table 4:** Variants identified via WES and selected as targets for mPCR experiments. Variants in bold were selected as targets for ddPCR assays.

**RT-6736**			
**Gene**	**Alteration**	**Effect**	**VAF**
***DDX3X***	**A1038C**	**Nonsynonymous SNV**	**0.542**
*ARMCX4*	G5670A	Synonymous SNV	0.474
*DDX49*	C1122T	Synonymous SNV	0.467
*NAA38*	C174T	Synonymous SNV	0.36
*ISLR*	C229A	Nonsynonymous SNV	0.322
*OR52K2*	C528T	Synonymous SNV	0.255
*FLOT2*	G547A	Nonsynonymous SNV	0.217
*LRRC66*	G1944A	Synonymous SNV	0.211
**RT-7418**			
**Gene**	**Alteration**	**Effect**	**VAF**
*TNS2*	G3243A	Synonymous SNV	0.821
*CYP27B1*	C667T	Nonsynonymous SNV	0.702
*CDH19*	C515G	Nonsynonymous SNV	0.413
*NOC4L*	G504A	Synonymous SNV	0.389
***SEMA5A***	**C2182T**	**Stopgain**	**0.353**
*CCL22*	73+2T>A	Splice variant	0.343
*LRRC6*	A959G	Nonsynonymous SNV	0.292
**RT-7421**			
**Gene**	**Alteration**	**Effect**	**VAF**
*ATRX*	6071_6072del	Frameshift deletion	0.763
*KIF19*	A1081G	Nonsynonymous SNV	0.571
***ENPP3***	**C512A**	**Nonsynonymous SNV**	**0.556**
*PIEZO2*	A1604G	Nonsynonymous SNV	0.481
*HNF4A*	C859T	Nonsynonymous SNV	0.446
*MYH11*	C577T	Stopgain	0.261
*PCLO*	C6498A	Stopgain	0.224
**RT-7860**			
**Gene**	**Alteration**	**Effect**	**VAF**
*USP26*	T1393C	Nonsynonymous SNV	0.833
***ULK2***	**A604G**	**Nonsynonymous SNV**	**0.743**
*HIPK4*	C1012T	Nonsynonymous SNV	0.419
*PDGFRB*	C3066T	Synonymous SNV	0.409
*TNPO3*	396-4G>C	Splice site variant	0.37
*ILDR1*	A925G	Nonsynonymous SNV	0.359
*CLDN19*	223+14C>T	Splice site variant	0.25

**Table 5:** Variant sequences detected in cfDNA via mPCR-amplicon sequencing.

RT-6736		RT-7418		RT-7421		RT-7860	
Amplicon	Allele Detected	Amplicon	Allele Detected	Amplicon	Allele Detected	Amplicon	Allele Detected
*DDX3X*	**MUT**	*TNS2*	WT	*ATRX*	**MUT**	*USP26*	WT
*ARMCX4*	WT	*CYP27B1*	WT	*KIF19*	**MUT**	*ULK2*	WT
*NAA38*	**MUT**	*CDH19*	WT	*ENPP3*	**MUT**	*HIPK4*	WT
*OR52K2*	WT	*NOC4L*	WT	*PIEZO2*	**MUT**	*PDGFRB*	WT
*FLOT2*	WT	*SEMA5A*	WT	*HNF4A*	**MUT**	*TNPO3*	WT
*LRRC66*	**MUT**	*CCL22*	WT	*MYH11*	WT	*ILDR1*	WT
		*LRRC6*	WT	*PCLO*	WT	*CLDN19*	WT

Overview of mPCR amplicon sequencing of cfDNA samples for the four cases tested. WT denotes that only the wild-type allele was detected, whereas MUT indicates that the variant allele was detected.

**Table 6:** Clinical data.

Case	Diagnosis	Grade	Presence of Metastases	ctDNA Detected
RT-7418	Well differentiated liposarcoma	1	None	No
RT-7860	Pleomorphic liposarcoma	2	None	No
RT-7707	Myxofibrosarcoma	3	Mets to lung	Yes
RT-7421	Undifferentiated pleomorphic sarcoma	3	Mets to lung	Yes
RT-6736	Osteosarcoma	3	Extensive mets to femur and retroperitoneum	Yes

## Data Availability

The data presented in this study are available upon written request.
